# A Remote Assessment of Anxiety on Young People: *Towards* Their *Views* and Their Different Pet Interaction

**DOI:** 10.3390/healthcare10071242

**Published:** 2022-07-03

**Authors:** Daniele Giansanti, Mariacristina Siotto, Giovanni Maccioni, Irene Aprile

**Affiliations:** 1 Centro Nazionale Tecnologie Innovative in Sanità Pubblica, Istituto Superiore di Sanità, 00161 Rome, Italy; giovanni.maccioni@iss.it; 2 Istituto di Ricovero e Cura a Carattere Scientifico Fondazione Don Carlo Gnocchi, 50143 Florence, Italy; msiotto@dongnocchi.it

**Keywords:** COVID-19, social distance, youth, anxiety, pet, mental health, eHealth

## Abstract

The lockdown was imposed in Italy on 9 March 2020 due to the COVID-19 outbreak. Restrictions severely limiting individual freedom were indispensable to protect the population and reduce virus diffusion. Italian people had never before experienced similar restrictions that undoubtedly tested psychological health. After 1 week, we developed an electronic survey to collect demographic data and information on the presence of pets and the type of interaction with them and to administer a self-assessment anxiety test. A total of 3905 subjects, pet owners and non pet owners, filled in the electronic survey; 652 (16.7%, mean age 21.6) of them were young subjects, adolescents, and university students. The study *first* showed the feasibility and success of the technological solution used, capable of providing, at a distance, structured information on the participants and quantitative data on the psychological condition. *Second*, it reported that 23.1% of the youths showed anxiety above an attention level during the lockdown, in line with other studies. *Third*, it indicated, based on the outcome of the self-assessment test, that the pet presence could have a positive effect in mitigating the psychological impact and encourage to continue and deepen these investigations. *Fourth*, it reported positive feedback from the participants on the procedure, found useful during the pandemic and for the post-pandemic future. The study highlights the importance of investing in these solutions based on mobile technology and useful both for mental health and to deepen the investigation of the impact of the pet presence on the human psychology.

## 1. Introduction

On 11 February 2020, the WHO [1] announced the name of the respiratory disease caused by the new coronavirus: COVID-19 in 2020. On 9 March 2020, in response to the growing pandemic of COVID-19 in the country, the Italian government imposed lockdown measures. The lockdown was never experienced before by Italian people. During the lockdown and the pandemic evolution, mobile technology (*mTech*) was crucial for tolerating the social distance imposed by the COVID-19 [2,3]. The *mTech* was useful (and is currently useful): to support teaching, work, and the relational activities [4,5]; to provide the *mobile health* and solutions for managing and following the spread of the pandemic [6,7,8].

### 1.1. The Mobile Technology and the Mental Health

Through *mTech* it is possible to monitor mental problems, providing specific and consolidated tests remotely to obtain objective measures [9], with the potential to inform structured mental health interventions [10]. The Zung self-assessment test for anxiety [11] and for depression [12] could be provided remotely sending interactive forms by means of electronic surveys developed, for example, by means of *Google forms*, *Microsoft forms*, *and Survey monkey* [13,14,15]. From a telemonitoring point of view, an analogy can be drawn between the self-assessment of glucose, heart rate, or oxygen saturation with the self-assessment of anxiety. In the first case, the remote measuring tools are the glucometer, the heart frequency meter, and the oximeter, which return numbers associated with the state of health related to diabetes and the cardiorespiratory apparatus. In the second case, the remote measuring tool is the psychological test (delivered through an electronic survey), which returns a number associated with the level of anxiety and, therefore, the level of psychological health. As for psychological health, the self-assessment of anxiety or depression through *mTech* can be useful to mitigate the detrimental impact of stressful events, such as, in this period, the COVID-19 pandemic. Behind the advantages of the massive use of *mTech* there are also disadvantages.

The opposite side of the coin of the vantages of *mTech*, already known and aggravated with the lockdown and subsequently, is that of a push towards *living online* with no social contacts, no motivation to go outside and contact people when everything can be done remotely from home. Previously, there was concern about the negative impact of excessive use of smartphones and tablets, especially among young people [16]. The risk ranges from postural problems, such as text neck, to distortions in the type of communicative interaction, without the three basic components (verbal, para-verbal, and non-verbal), up to a real addiction with a psychological impact on the person [17]. All this during the lockdown and immediately after it got worse [18].

### 1.2. The Psychological Impact of the Pet Presence in Stressful Situations in Young People

Many factors can affect an individual’s psychology during a period of acute stress, such as the lockdown, with an aggravating or mitigating effect [19].

Previous, several studies reported that emotional and physical health could benefit from pet presence and interaction [20].

Many studies have shown that the presence of pets can mitigate the impact of stressful situations in young people [21,22,23,24]. These studies showed that the presence of pets can psychologically help young people [21] and has a major impact on students [12,13,14,15,16,17,18,19,20,21,22,23,24]. Other studies have highlighted the need to quantify this mitigating effect through specific procedures, complaining that the positive impact of companion animals is difficult to calculate, even if it is a simple co-presence [25,26]. Another study highlighted a new way of applying the AAT [27]. We are today, in fact, assisting to an increasing interest in both animal-assisted therapy (AAT) and the pet quality of life and health. The increasing interest in the pet quality of life and health is a direct consequence of the recognition of its contribution to the society. Today, according to the new central position of the pet, the approach must be revised in a more general and bidirectional approach embedding the assessment of the health benefits contemporary for the two actors, human and pet [25,27].

We also did a search on Pubmed with the composite key:

*(animal assisted therapy[Title/Abstract]) AND ((anxiety[Title/Abstract]) OR (depression[Title/Abstract]) OR (mental health[Title/Abstract]) OR (psychological disorder[Title/Abstract])) Filters: Review* [28].

It returned 34 reviews that confirm the wide area of use of AAT from a clinical point of view as a complementary therapy. Four highlight a positive impact of AAT on minimizing anxiety in subjects with and without neurological diseases [25,29], with minimal risk [30] and with potential also on other psychological problems such as depression [31].

The results are also in line with other studies focused on the advantages of AAT when there are important situations of constraint [32] that, in a certain sense, are similar to lockdown. The study by Villafaina-Domínguez [32] reviewed the ATT, applied in concrete dog-assisted intervention introduced in prisons to reduce recidivism as well as to improve the wellbeing of prisoners. The study concluded that dog-based animal-assisted therapy may improve anxiety, stress, recidivism, and other social variables in male or female inmates.

### 1.3. The Impact of the Pandemic on the Youth’s Psychology

There was a concern about the lockdown’s effects on the population. The young people, representing the most active population with the greatest need for sociality, were forced into an unnatural condition for them at home (giving up the moments of sociality) and were forced to carry out distance learning and distance interactions. Some studies measured the anxiety and depression during the COVID-19 pandemic and the impact on the lockdown [33]. The young part of the population (adolescent and university students) has been particularly psychologically strained by the restrictions [33,34]. The impact of the lockdown on anxiety and depression has been faced, for example, in [19]. The coronavirus disease 2019 (COVID-19) pandemic presented an opportunity to explore the role of animals as sources of emotional and physical support during a period when most of the population experienced social and environmental challenges. Several studies have addressed the impact of the presence of pets during the pandemic [35,36,37,38,39,40,41,42,43,44,45,46,47,48]. These studies addressed different aspects and led to different results. For example, it was shown in [46] the beneficial impact on human psychology. The study also addressed the problem from the point of view of the quality of life of the animal showing that pets showed signs of behavioral change that were consistent with stress. Other studies have shown how excessive attachment to the animal can increase mental distress [47]. Contrary to expectations, the findings suggest that during a specific situation such as a pandemic, a high attachment to pets may contribute to an increased burden among owners and contribute to poorer quality of life [47]. The study reported in [35] showed the importance of the environment of life. For participants in rural and semi-urban areas, living with a dog was associated with lower anxiety; for participants in urban areas, living with a dog was associated with higher anxiety [35]. Attachment to one’s companion animal was found to be a strong predictor of mental wellbeing, with higher bonds of attachment associated with higher levels of depression, loneliness, and lower levels of positive experience [36]. Four main themes related to the human-animal interactions during the COVID-19 lockdown phase were identified as strategic in [37] for investigations in this field: the positive impact of animal ownership during the COVID-19 lockdown (e.g., amelioration of wellbeing and mental health), concerns relating to animal ownership during the COVID-19 lockdown (e.g., concerns over animals carrying the COVID-19 virus), grief and loss of an animal during the COVID-19 lockdown, and the impact of engaging with non-companion animals during the COVID-19 lockdown.

#### Purpose of the Study

The aim of the study was:

- To remotely administer, through the *mTech*, an electronic survey: to collect demographic data; to administer a psychological test consolidated in literature (Zung test [49,50,51]) for the measurement of the anxiety in young people; to collect information on the co-presence and interaction with pets.
- Collect structured feedback and views from the participants also in prospective use.

## 2. Methods

### 2.1. Participants and Procedure

#### 2.1.1. The Tool: The Structure

The tool included three sections: (a) a section dedicated to the information of the participants, asking for their consent to the survey, the information related to demographic data (sex, age, school level, country), and the presence or absence of previous psychological problems (people with previous mental problems were excluded); (b) a section dedicated to the relationship with the pet (only for pet owners); (c) a section dedicated to the anxiety self-assessment Zung test [49,50,51]; (d) a section with graded and open questions asking opinions on the methodology. The section dedicated to the pet owners was both for dog and cat owners. We have excluded, as a structured proposal to be selected, the horse, which does not live at home, and other animals, such as the rabbit and the ferret, which are not very common and which are not recognized as demonstrating a large contribution as psychological support. The participants, however, had free fields for comments and opinions to possibly introduce these animals. A feedback form with graded and open questions asking opinions on the methodology was submitted along with the tool.

#### 2.1.2. Submission and Participants

The only prerequisite that we set ourselves to limit the articulations of the study, and the ethical implications, was to include participants who have never had psychological problems. In fact, the inclusion of participants with psychological problems would have involved a specific authorization procedure that would have prolonged the approval times, making them incompatible with the timeliness of the application foreseen by the emergency. We also decided to focus on students not yet involved in the world of work in order not to add another confounding factor to the study (work). The submission took place through social media, such as Facebook, Linkedin, Twitter, Instagram, and Whatsapp, association sites of animal lovers, and in general, a peer-to-peer dissemination.

We have also encouraged both the spread of the electronic survey and the support in filling out those who are less familiar with digital technology (also strongly specifying this in the electronic survey introduction).

Table 1 shows the demographic characteristics of the participants in the study: all students attended secondary schools or universities, and none had previous psychological problems. The table shows these participants divided into the two pet owner and non-owner groups. Some pet-owners, as shown in the table, had more pets.

### 2.2. Measures

The original survey is in Italian and is closed and no longer accessible.

We have translated a version from Italian into English for these editorial purposes. The link to the interactive tool is the following: https://forms.office.com/Pages/ResponsePage.aspx?id=DQSIkWdsW0yxEjajBLZtrQAAAAAAAAAAAAZAAOUXdFhUQ0gxM1U0MkhTNVNUSEQ0WE04OTlWTUY2Uy4u (accessed on 3 June 2022).

The survey considered various parameters for the collection and evaluation of information, some of which were used in this work, and others will be further explored later. The survey was submitted to a large sample (much larger than that investigated in this proposal). The following parameters were considered related to the submission rate: total submission, total number of people who opened the survey but did not participate, the total number of those who decided not to perform the anxiety test, the total number of people who participated in all parts of the survey. As for the specific submission to the sample in Table 1, they were considered: age, gender, student status, possession of one or more dogs, and possession of one or more cats. In the Zung test, the subject is asked to carefully read 20 listed sentences and choose the answer that better describes the situation in the last week experienced. The answer to each question allows a graded evaluation with a score ranging from 1 to 4. The scores obtained for each question are added together. A threshold (*Z th*) = 40 is fixed to identify a moderate level of anxiety, i.e., a level of attention. A value of the Zung test (*Z score*) under or equal to the *Z th* indicates low or very low anxiety. With a *Z score* above the *Z th*, the anxiety is moderate or high. We refer to this *Z th* to identify the participants with a level of attention of anxiety.

The timeliness of the application of the Zung test is vital. The perception indicated in each item must refer to the latest week. We aligned the administration of the tool to exactly one week after the lockdown, and we kept the survey open for a very few days. We, therefore, also wanted to measure the response speed from the submission.

We also asked, by means of a separated form, an opinion using graded questions and open questions (for comments) to have feedback on the administration process. We established a six-level psychometric scale for the graded questions. It is possible, therefore, to assign a minimum score of one and a maximum of six with, therefore, a theoretical mean value (TMV) of 3.5. We can refer to the TMV for comparison in the analysis of the answers. An average value of the answers below TMV indicates a more negative than positive response. An average value above TMV indicates a more positive than a negative response. The outcome of the open questions was investigated qualitatively.

**Figure 1:**
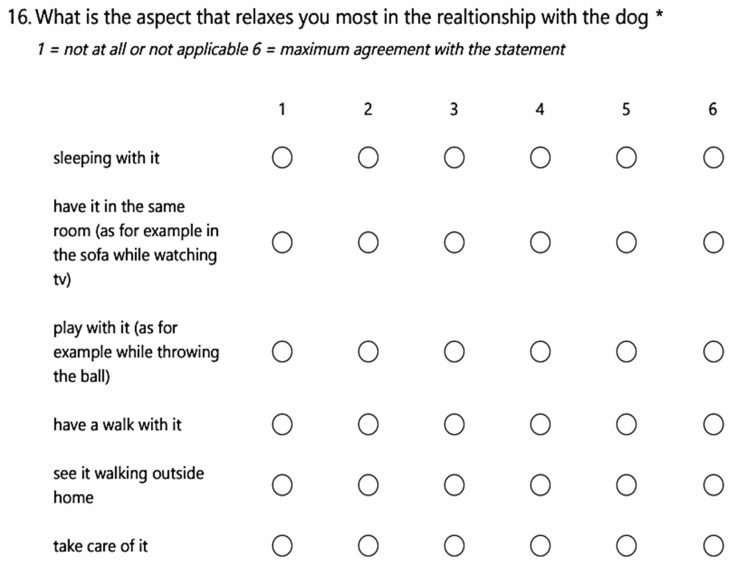
Tool Section (Likert) dedicated to the questions about the interaction with the dog. *: means compulsory.

In the survey, there are also Likerts (for example, question 16, Figure 1) available for further data mining. We established a six-level psychometric scale for the Likert as the graded questions.

### 2.3. Statistics

We used the Smirnov–Kolmogorov test for testing the normality of the distribution of both age and anxiety, as it is preferable for not small samples, such as ours [52]. The null hypothesis for the distribution of anxiety was that our data on anxiety followed a normal distribution. The null hypothesis for the distribution of age was that our data on anxiety followed a normal distribution. We applied the Student *t*-test (with a *p* < 0.01 for the assessment of the significance) after the check of the normality in the data distribution when comparing the mean anxiety scores in the two groups in Table 1. The null hypothesis was that there was no difference between the mean anxiety scores. We applied the χ^2^ test (with a *p* < 0.01 for the assessment of the significance) in the frequency analysis to assess the significance of the prevalence of anxiety (difference in the percentage of anxious subjects between the two groups). The null hypothesis was that there was no difference between the frequencies of the anxiety. The software SPSS version 24 (IBM, Armonk, NY, USA) was used in the study.

## 3. Results

The results were organized into four paragraphs. The first paragraph reports the results on the timeliness of the test administration. The second paragraph reports a statistical analysis of significance, introductory to tests, then applied to the analysis of the outcome. The third paragraph is dedicated to the central analysis of the study, i.e., the analysis of the participants’ anxiety. The fourth paragraph reports the analysis of the participants’ feedback on the method and the perceptions.

### 3.1. Submission and Response Speed Rate

The electronic survey was sent on 15 March 2020. Subjects began responding on 16 March, exactly one week after the start of the *lockdown* in Italy (which occurred on 9 March 2020). The tool remained active until March 25. In total, 91.74% of responses were obtained in the first 4 days; all samples terminated the survey within a week. A total of 100% of the sub-sample of the young subjects terminated the survey in the first three days. This is important as it indicates a high concentration of responses around a week after the traumatic event, taken into consideration by the Zung test. We sent 4993 electronic surveys; 389 subjects did not give their consent or could not be included because of previous psychological problems; 799 subjects did not fill in the anxiety test; 3905 anonymous subjects with and without a pet (dog and/or cat) filled in completely the electronic survey (age: 14–77 years; average age 44.7 years; 1913 males; 1992 females).

A sample of 652 (16.7%) of them were young subjects (age: 14–29) selected in this study, divided into two groups based on the pet ownership (Table 1).

### 3.2. Preliminary Test of Statistical Significance

Preliminarily to the analysis, we applied the selected tests to verify the normality of the data. We tested the distribution of age for the two samples, pet owners and not pet owners, by the Smirnov–Kolmogorov test of normality, which is suitable for large samples such as ours. The null hypothesis was that our data followed a normal distribution. We achieved *p* = 0.53 for the pet owners and *p* = 0.51 for the not pet owners. Because *p* > 0.05, we accepted the null hypothesis. We therefore faced a normal distribution in the two samples. We also tested the *distribution of the Z score* for the two samples by the Smirnov–Kolmogorov test of normality. The null hypothesis was that our data followed a normal distribution. We achieved *p* = 0.50 for the pet owners and *p* = 0.52 for the not pet owners. We were therefore faced with a normal distribution.

### 3.3. Outcome from the Zung Test

**Figure 2:**
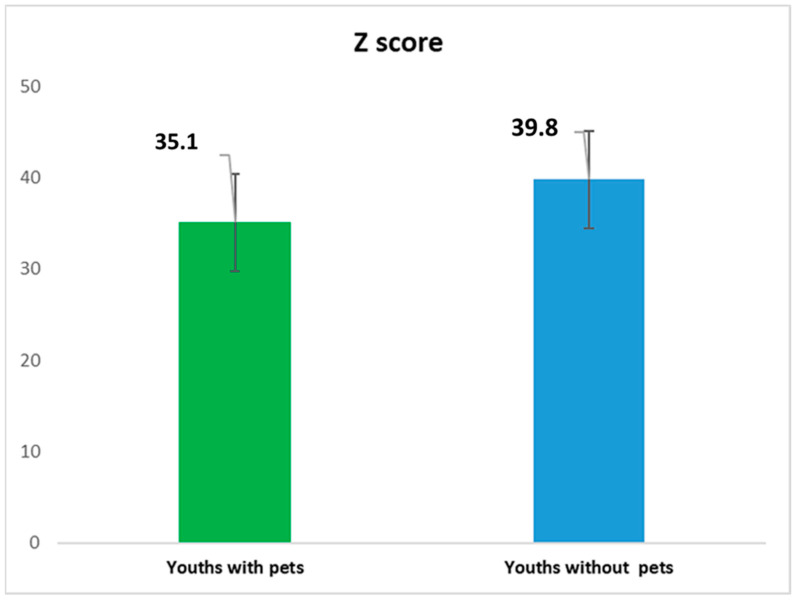
The Z score both for youth pet owners and for youths without pet.

We analyzed both the frequency of anxiety (*Z score* ≥ *Z th*) and the averaged values of the *Z score*. In the first case, we applied the χ^2^ test, and in the second, the Student *t*-test. In total, 150 participants showed a *Z score above the Z th*, an attention level for anxiety (anxiety for the sake of brevity in the following) during the lockdown (23.1%). We applied the χ^2^, which showed a very high significance on the difference (*p* < 0.01). Figure 2 shows the average value of the *Z scores* in the two groups. The group of pet owners showed a lower value. We applied the Student *t*-test. The tested hypothesis was the significance of the difference between the averaged values between the two groups. The Student *t*-test showed a highly significant difference (Student *t*-test, *p* < 0.01).

**Figure 3:**
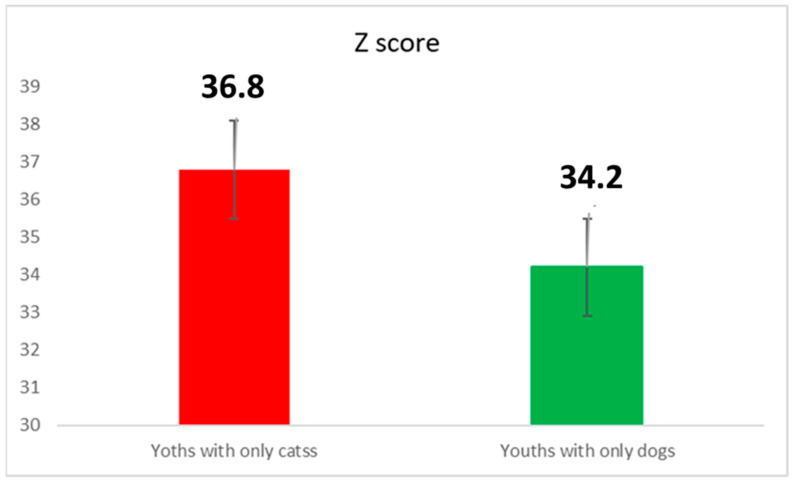
The Z score both for youth cat and dog owners.

We further deepened the analysis by comparing the *Z score* between owners of cats only and owners of dogs only. The group of dog owners showed a lower value when compared to the cat owners (Figure 3). We applied the Student *t*-test. The tested hypothesis was the significance of the difference between the averaged values between the two subgroups. The student *t*-test showed a high significant difference (Student *t*-test, *p* < 0.01).

### 3.4. Feedback from the Participants

We analyzed the feedback obtained through open and graded questions. All the answers to the graded questions, highlighted a high degree of acceptance (score > 5) of the methodology as regards all the proposed parameters: reliability, practicality, clarity, usefulness, potential in the health domain during the pandemic, and prospects in the health domain in the post-pandemic future.

Some selected comments are reported in Table 2. The selection highlights: (a) the appreciation of the method both as a tool for tele-mental health and as a tool to highpoint the importance of the pet as psychological support; (b) in some rare cases, both the pet presence and the telemonitoring approach are not appreciated.

**Figure 4:**
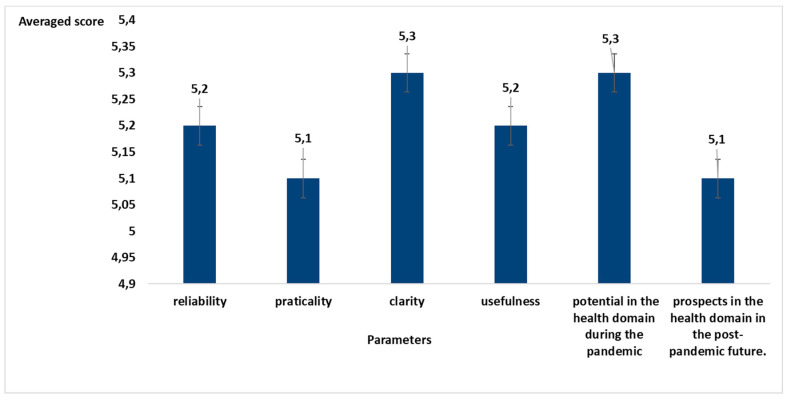
Average score for the different parameters proposed to assess the opinion of the participants.

A further analysis thematically deepened what emerged in the free comments. Figure 4 reports the outcome. The numerical summary of the comments that reported more than three occurrences is shown.

The thematic analysis shows that:

1. The dog is recognized as having the added value of stimulating physical activity during the lockdown. In fact, in Italy, the dog represented a passport to go out for a walk during the most important restrictions.
2. The dogs and the cats are recognized as comparable contributions to wellbeing.
3. The questionnaire was found to be very useful.

**Figure 5:**
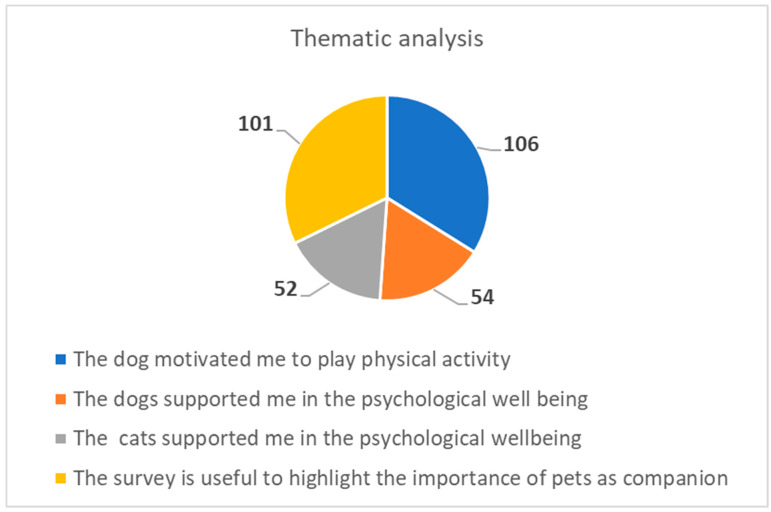
Outcome on the thematic analysis on the comments.

Point (1), if read together with the graph shown in Figure 5, seems to give interpretative support to the lower anxiety values found in dog owners.

## 4. Discussion

The outcome has *four points of view*, which are interrelated.

The *first point* of view is related to the administration of an electronic telemonitoring tool for the collection of data (also relating to pets) and for the administration of an anxiety test.

The *second point* of view focuses on the administration of a self-assessment tool for anxiety.

The *third point* of view is on the analysis of the differences in the anxiety between pet owners and non-pet owners.

The *fourth point of view* passes the word to the participants and collects structured and free-open feedback on the procedure and on prospects.

The *first point of view* highlights the feasibility and effectiveness of administering these telemonitoring surveys, including through *mTech*. The results show that 100% of the young subjects terminated the survey in the first three days, and the data were, consequently, all available centrally. The entire sample terminated the survey within a week. Such timeliness (which, as it has been illustrated, was also important for synchronizing the Zung test) and ease of administration makes these tools very useful at the government level as sensors on the population.

In addition to the high acceptance, measured in feedback from the participants (*fourth point of view*), we can consider various experiences demonstrating the potential of these tools and corroborating their usefulness. In the U.S., for example, an electronic tool has been proposed and submitted using Facebook for this purpose. This tool has been continuously adapted to the phases of the pandemic [53]. A recent review highlights how these tools have gradually been used in different countries during the pandemic [54].

The *second point of view* points out the importance of remotely monitoring the psychological condition of young people. This is in line with many studies conducted on the psychological impact of pandemic restrictions on young people of a wide range of ages, including both young adolescents and students in advanced university courses [33,35,55,56]. These studies have shown how the restrictions could cause psychological problems, including anxiety. In line with the solutions proposed in [53,54], we developed an electronic survey to investigate this. The part of the survey dedicated to the anxiety test made it possible to highlight, as a first important and troubling result, the very high anxiety prevalence rate (23.1%) among the youths during the COVID-19 outbreak in line with other studies [33,35,55,56]. The usefulness and practicality of this method of administering psychological tests through population surveys, in addition to the high acceptance by the participants (*fourth point of view*), is reflected in other studies conducted during the pandemic, such as in Qiu J et al. [57].

We thought about the psychological problems of young people [33,35,55,56], we shared the need to explore solutions to mitigate the distress they are experiencing [58], and we investigated the potential of pets [22,23,24] in line with other studies that investigated the impact of the pet during the pandemic [35,36,37,38,39,40,41,42,43,44,45,46,47,48].

This third *point of view* is in line with other studies that indicate the usefulness of the pet in mitigating some psychological problems due to isolation during the pandemic, for example, with (I) the study by Gaifoner et al. [38] indicating that pet owners reported significantly better coping self-efficacy, significantly more positive emotions, and better psychological wellbeing; (II) the study by Muller et al. [42], highlighting that adolescents with pets reported spending more time with their pets during the pandemic, and frequently reported pet interactions as a strategy for coping with stress; (III) the study by Ratshen et al. [48] highlighting that the human–animal bond is a construct that may be linked to mental health vulnerability in animal owners and showing that animal ownership seemed to mitigate some of the detrimental psychological effects of COVID-19 lockdown; (IV) the study by Bowen et al. [46] showing that the quality of life of owners was strongly influenced by the lifestyle and emotional effects of the confinement and that pets provided them with substantial support to mitigate those effects.

This *third point of view* indicated a higher level of anxiety among the youths not owning pets than among youths not owning pets. Differences were also found between the cat-only and dog-only groups. Dog-only owners showed lower levels of anxiety as measured by the test. This, albeit with the limitations of the study listed below (which do not yet allow us to pronounce ourselves firmly on the investigated therapeutic effect of the pet to mitigate anxiety), suggests to stakeholders to continue investigating this important issue with these methods.

The latest, *fourth point of view* highlights positive feedback from the participants. The tool was scored highly reliable (averaged score > 5, with six levels of scoring), practical, clear, useful, with an important potential in the *health domain* during the pandemic and prospects for the post-pandemic future. The *fourth point of view* also highlighted, through a thematic analysis of free comments proposed, some important perceptions on pet owners. The dog, for example, was recognized as having the added value of stimulating physical activity during the lockdown. This seems to be able to justify the lower levels of anxiety found in dog owners.

### Limitations

Further investigations are needed to evaluate the impact of our issues on pet ownership, for example, the environment, both as a place of life (rural, urban) [35] and as a family structure. The study was configured with an emergency set-up to allow the project to start no later than a week after the announcement of the Italian lockdown. The experience of lockdown was new and never tried before. It was not possible to predict (and therefore include) a priori all the environmental and social factors. To avoid specific authorizations (which would have required more time and which would have compromised the timeliness), the project did not include participants with psychological problems. The bias from the digital divide (which in any case is low in young people) was minimized (not eliminated) with solutions of encouragement to support the less accustomed to *mTech*, yet successfully tested in [59]. Electronic surveys, despite having numerous advantages as strong sensors on the population, are commonly used in the science of life applications and, in general, are subject to limitations. These limitations have been already introduced in some studies cited and conducted at a national level [53,54,59], such as the “willingness” and the type of administration that includes the participants in the study with a “fish on the pile procedure”, that we tried to compensate designing and applying a robust statistic.

## 5. Conclusions

The COVID-19 pandemic represented an important opportunity to improve the use of *mTechs* and broaden their boundaries of use. In our proposal, we used electronic survey technologies to explore on young people (a) the impact of anxiety during the lockdown restrictions, (b) the seemingly mitigating effect of pets during these restrictions, (c) the effectiveness of the telemonitoring tool, (d) the acceptance of the proposed telemonitoring solution, and (e) the perceptions of the interviewed.

The outcome: *first*, in line with other studies, the study highlighted the presence of anxiety during the lockdown on young people. *Second*, it projected the idea that the pet presence could have a positive impact in mitigating the psychological impact and encourage to continue and deepen these investigations. *Third*, it showed the feasibility and success of the technological solution, capable of providing, at a distance, structured information on the participants and quantitative data on the psychological condition (*Z score*) and perceptions.

### Three Important Reccomandations Emerge

The *first recommendation* is the importance of motivating stakeholders to invest resources in these solutions, appreciated by the participants and considered useful during the pandemic and in the post-pandemic future. The *second recommendation* relates to the importance of investing in research solutions that make it possible to highpoint the contribution of the pets in mitigating the psychological impact of stressing factors. The *third recommendation* is the potentiality of remote administration of the psychological investigations.

## Figures and Tables

**Table 1:** Demographics characteristics.

Young Participants	Students	Psychological Problems	N	Pet Owned	
*Pet owners* Age between 14 and 29 years; 50.7% males (mean age 21.7 years; maximum age 29 years); 49.3% females (mean age 21.4 years; maximum age 29 years)	All	No	370	One or more dogs	179
				One or more cats	124
				Cats and dogs	67
*Not pet owners* Age between 14 and 29 years; 50.5% males (mean age 21.5 years; maximum age 29 years); 49.5% females (mean age 21.6 years; maximum age 29 years)	All	No	282	None	

**Table 2:** Selected open answers.

Comment
***I do not appreciate the administration of psychological tests remotely, I think that this type of evaluation must always done in presence even in periods in which social distancing is in force.***
***I think that the Italian government could use these methods for targeted assessment campaigns on categories of subjects exposed to psychological risks, but I think that familiarity with smartphones and tablets should be increased to broaden the audience.***
***The use of psychology in Italy is still very limited. There are countries where the psychologist is requested with the same frequency as the family doctor. Surely reaching the citizen with these tools could help to bring out hidden problems.***
***I really appreciated this survey both for its aspects related to anxiety and because it tried to highlight the importance of pets that in my opinion are our angels*** *and are helping us a lot in this moment of emergency*
*Pets are a great help that is often underestimated and little appreciated as psychological support. **All the initiatives that support the role and importance of pets are well***
*I took the test. I did not know it and I found it very useful. **I don’t have a dog or a cat but those who have one tell me that in this moment of emergency they feel particularly helped by the proximity of this animal. I’m thinking of getting one and the survey has given me a boost in this direction.***
***I am very convinced that pets reduce anxiety and depression. These measures are useful to politicians because they realize that they need to help with economic incentives those who have pets and those who cannot afford them, such as the elderly with a low pension.***
*I am allergic to cat and dog hair and I think all this hype around the usefulness of pets is wrong. **We should try to be closer between us humans so both anxiety and stress decreases.***
*It seems to me that the proposed system, if I have understood well, allows for telemedicine. **Always, if I understand correctly, the dog in this case is the therapy against anxiety, a bit like insulin against high blood sugar (which I have), the psychological test measures anxiety like the blood glucose machine is used for measure sugars** [].*
*I really liked the survey that, **I think, you developed in a hurry to face the emergency. In the future, however, I suggest that you include other parameters relating to the home or family or other disturbing factors** in the test*

## Data Availability

Not applicable.
